# Nuclear localisation of a fish-derived ice-binding protein and related transcriptional changes in *Caenorhabditis elegans*

**DOI:** 10.1016/j.bbrep.2026.102771

**Published:** 2026-09-02

**Authors:** Saki Nakamura, Kazuki Ozawa, Kotaro Ozaki, Yoichi Shinkai, Masahiro Kuramochi

**Affiliations:** a Graduate School of Science and Engineering, Ibaraki University, Hitachi, 316-8511, Japan; b Cellular and Molecular Biotechnology Research Institute, National Institute of Advanced Industrial Science and Technology (AIST), Tsukuba, 305-8566, Japan; c Graduate School of Frontier Sciences, The University of Tokyo, Kashiwa, 277-8561, Japan

**Keywords:** Ice-binding protein, NfeIBP6(A20I), *C. elegans*, Nuclear localisation, Gene expression

## Abstract

Ice-binding proteins (IBPs) are functional molecules that enable organisms to survive severe cold by binding specifically to ice crystals and suppressing their growth and recrystallisation. Although their primary role has traditionally been viewed as antifreeze activity, recent studies have suggested additional cell-protective functions, including possible effects on membrane stability. Heterologous expression of the fish type III IBP NfeIBP in *Caenorhabditis elegans* has previously been shown to increase tolerance to cold and freezing stress. However, the understanding of the intracellular localisation of IBPs in vivo and their effects on gene expression remains limited. Here, we examined the intracellular localisation and associated cellular effects of the fish-derived IBP variant NfeIBP6(A20I), which is known to confer strong cold tolerance in *C. elegans*. An NfeIBP6(A20I)–wrmScarlet fusion protein expressed in body wall muscle cells was detected in both cytoplasmic and nuclear regions and partially overlapped with the nuclear marker NLSsfGFP, indicating its presence within nuclear compartment. Expression of NfeIBP6(A20I) did not cause detectable abnormalities in the physiological traits examined. Transcriptomic analysis further revealed biased changes in gene expression, with substantially more downregulated genes than upregulated genes. The downregulated genes were significantly enriched in biological processes related to cuticle development and immune response. These findings indicate that NfeIBP6(A20I) exhibits previously unrecognised intracellular behaviour in *C. elegans* and suggest that the functional scope of IBPs may extend beyond their conventional role in ice binding.

## Highlights

• Fish-derived NfeIBP6(A20I) localises to the nucleus in *C. elegans.*
• NfeIBP6(A20I) is associated with biased transcriptional changes.
• NfeIBP6(A20I) expression lacks detectable physiological toxicity.

## 1 Introduction

Organisms inhabiting extremely cold environments have evolved diverse cryoprotective strategies to mitigate damage caused by freezing. Among the key molecules involved in these adaptations are ice-binding proteins (IBPs), a specialised class of proteins that bind specifically to the surface of ice crystals, thereby suppressing their growth and recrystallisation [[1], [2], [3]]. Since their initial discovery, IBPs have been identified across a wide range of taxa, including fish, plants, insects and fungi [[4], [5], [6], [7], [8], [9], [10]], and are also referred to as antifreeze proteins (AFPs) or thermal hysteresis proteins (THPs).

Historically, studies of IBPs have focused mainly on their antifreeze activity in extracellular fluids [[11], [12], [13]]. However, increasing evidence suggests that the functional scope of IBPs extends beyond simple ice crystal modulation. In addition to interacting with ice, IBPs have been proposed to act on biological membrane structures such as cell membranes and lipid bilayers, thereby contributing to membrane stability under low-temperature stress [[14], [15], [16], [17]]. Indeed, heterologous expression studies in the model nematode *Caenorhabditis elegans* have shown that IBP expression can increase survival not only during freezing but also under nonfreezing cold stress [[18], [19], [20], [21]].

One particularly informative model is NfeIBP, a type III IBP derived from the notched-fin eelpout *Zoarces elongatus* [22]. The activity of NfeIBP is highly sensitive to specific amino acid substitutions; for example, the NfeIBP6 isoform exhibits markedly altered ice-binding activity depending on the residue at position 20. In particular, the A20I variant, in which alanine is replaced by isoleucine, shows enhanced ice-binding activity [23]. Previous studies have shown that transgenic *C. elegans* expressing NfeIBP6(A20I) in body-wall muscle display strong cold tolerance [20], suggesting a relationship between antifreeze activity and organismal protection even under nonfreezing low-temperature conditions.

Despite these advances, the intracellular behaviour of IBPs in animal systems remains poorly understood. Fish-derived IBPs are generally thought to be secreted into extracellular fluids, where they help protect body fluids from freezing [1,24]. In contrast, some plant-derived IBPs have been reported to localise intracellularly, including within nuclear regions, although the physiological significance of this localisation remains unclear [25]. Whether IBPs show similar intracellular localisation in animal cells and whether such localisations are associated with changes in cellular state or gene expression remain largely unknown. Therefore, elucidating the spatial distribution of IBPs in vivo is important for understanding the broader functional roles of these proteins.

In the present study, we focused on NfeIBP6(A20I), a variant known to confer strong cold tolerance, and examined its intracellular localisation in *C. elegans*. By combining high-resolution confocal imaging with transcriptomic profiling by RNA-seq, we investigated the physiological effects of IBP expression and its association with changes in host gene expression. Our findings provide new insight into the intracellular behaviour of IBPs and suggest that their functional scope may extend beyond conventional ice binding.

## 2 Materials and methods

### 2.1 Plasmid construction and transgenic strain generation

The *myo-3* promoter was used to express IBP in the body wall muscles of *C. elegans*. Notched-fin eelpout (*Zoarces elongatus Kner*) possesses multiple IBP isoforms, among which the antifreeze activity of the sixth isoform markedly differs depending on substitution at the 20th amino acid residue (alanine, A20). In this study, the A20I variant, which exhibits the highest ice-binding activity, was used [20,23]. The plasmid DNA encoding this variant and the fluorescent proteins used for analysis were prepared as described in our previous studies [18,20]. For nuclear localisation analysis and control experiments, *myo-3p::NLS::sfGFP* and *myo-3p::wrmScarlet* constructs were generated. These constructs were based on pPD95.79 (a kind gift from Andrew Fire), and synthetic *NLS::sfGFP* or *wrmScarlet* sequences were inserted into fragments containing the *myo-3* promoter at the *Nhe*I/KpnI and KpnI/EcoRI sites, respectively.

Transgenic *C. elegans* were generated by standard microinjection [26]. The strains used in this study were as follows: CMS61 Ex[*myo-3p::wrmScarlet::NfeIBP6(A20I)* (15 ng/μL)], CMS69 Ex[*myo-3p::wrmScarlet* (60 ng/μL)], CMS76 Ex[*myo-3p::wrmScarlet::NfeIBP6(A20I)* (15 ng/μL); *myo-3p::NLS::sfGFP* (80 ng/μL)], CMS79 Ex[*myo-3p::Venus::NfeIBP6(A20I)* (30 ng/μL); *myo-3p::NLS::wrmScarlet* (20 ng/μL)], CMS97 Ex[*baf-1p::sat2* (30 ng/μL); pPD49.26 (50 ng/μL); *myo-3p::wrmScarlet::NfeIBP6(A20I)* (20 ng/μL)], and CMS98 Ex[*baf-1p::sat2* (30 ng/μL); pPD49.26 (50 ng/μL); *myo-3p::wrmScarlet* (20 ng/μL)].

### 2.2 Confocal imaging of intracellular IBP localisation

Adult worms were anaesthetised in M9 buffer containing 50 mM sodium azide and mounted on 1.5% agarose pads. Images were acquired using an inverted confocal microscope (A1; Nikon, Tokyo, Japan) equipped with a 60× objective lens. Image acquisition and analysis were performed using NIS-Elements C, NIS-Elements C-ER, and Fiji (ImageJ distribution). DIC, red fluorescence (*wrmScarlet::IBP*), and green fluorescence (*NLS::sfGFP*) images were obtained. Merged images were generated using Fiji.

### 2.3 Toxicity-related assays

For the survival assay after exposure to 5 °C, well-fed adult worms were used. Worms were cultured from the egg stage to adulthood at 24 °C on nematode growth medium (NGM) plates seeded with *Escherichia coli* OP50 as a food source. Thirty to forty adult worms were transferred to assay plates. The worms were then incubated at 5 °C for 24 h, after which the plates were returned to room temperature (RT). Two hours after recovery at RT, the numbers of live and dead worms were scored based on their responses to gentle tactile stimulation. Thrashing behaviour was assessed by placing a single adult worm in M9 buffer and counting body bends for 1 min. For the muscle cell nucleus assay, transgenic worms expressing NLSwrmScarlet in body wall muscle cells were used. The number of muscle cells was estimated by counting the number of fluorescent nuclei. As *C. elegans* body wall muscle cells are mononucleated, counting fluorescent nuclei allows direct quantification of cell number. Worms were mounted on 2.0% agarose pads in 5 μL M9 buffer containing 50 mM sodium azide. Images were acquired using an inverted fluorescence microscope (IX71; Olympus, Tokyo, Japan) with a 40× objective lens, and the nuclei were counted manually. For the egg-laying assay, a single adult worm was transferred to a freshly seeded NGM plate, and the number of eggs was counted after 24 h. The worm was then transferred to a new plate, and the procedure was repeated for an additional 24 h. The total number of eggs was calculated over two days. For chemotaxis assays, approximately 50–100 adult worms were collected, washed in M9 buffer, and placed at the starting point on assay plates. Benzaldehyde (Bz) was used as the test compound: undiluted for high concentration and diluted to 0.001% in ethanol for low concentration. The test compound and the solvent control (ethanol) were spotted (5 μL each) at opposite ends of the plate. After 30 min, the worms in each region were counted manually. The chemotaxis index was calculated as (A − B)/(A + B), where A and B represent the number of worms on the test and control sides, respectively.

All the data were statistically analysed using Student's *t*-test.

### 2.4 RNA-seq analysis

RNA-seq analysis was performed using NfeIBP6(A20I)-expressing transgenic worms and wrmScarlet-expressing worms as controls. Worms were maintained on NGM plates supplemented with 100 μg/mL nourseothricin. Approximately 400 synchronised day-1 adults were collected and homogenised using QIAzol Lysis Reagent, and total RNA was purified using the RNeasy Mini Kit. Six RNA-seq libraries were initially prepared, comprising three control and three NfeIBP6(A20I)-expressing samples. Following quality control, one control sample and one NfeIBP6(A20I)-expressing sample were excluded from downstream differential expression analysis. The remaining four libraries, comprising two biological replicates per group, were used for the final analysis. Poly(A)-selected mRNA libraries were prepared and sequenced using 150-bp paired-end reads, generating approximately 5 Gb per sample. Reads were mapped to the *C. elegans* reference genome (WBcel235), and gene-level counts were obtained using featureCounts. Differential expression analysis was conducted using DESeq2 in R, with an adjusted p value < 0.05 as the significance threshold. Genes with adjusted p values < 0.05 were considered to be differentially expressed and were classified as up- or downregulated based on the sign of the log2-fold change. Volcano plots were generated using R and Python. Gene Ontology (GO) enrichment analysis was performed using clusterProfiler, focusing on biological process terms, with Benjamini–Hochberg correction applied.

## 3 Results

### 3.1 A fish-derived IBP shows nuclear localisation in *C. elegans* muscle cells

**Fig. 1:**
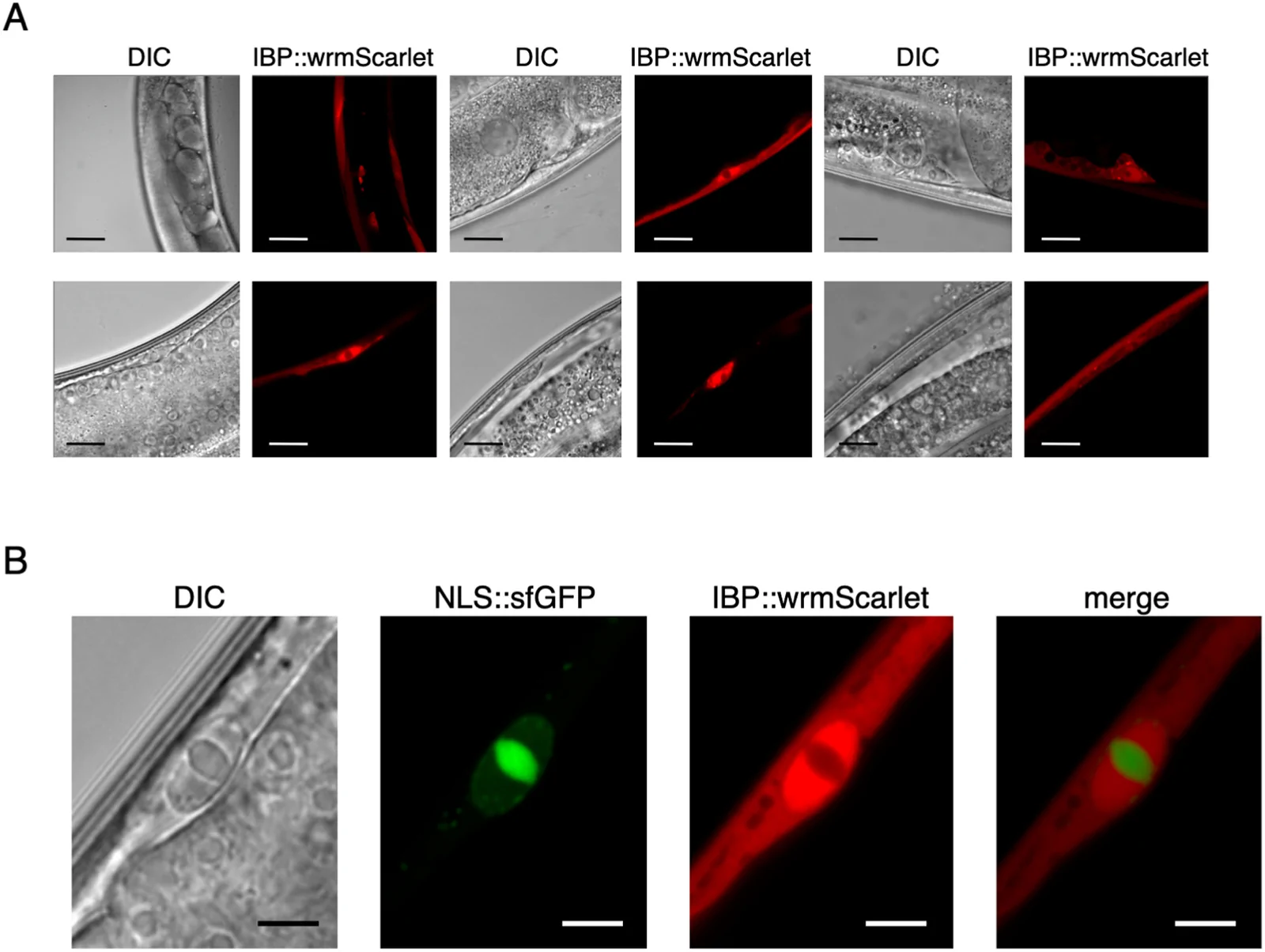
Subcellular distribution of NfeIBP6(A20I) in *C. elegans* muscle cells. (A) Representative confocal images showing the localisation of NfeIBP6(A20I)wrmScarlet in body wall muscle cells. DIC and corresponding fluorescence images are shown. The scale bar is 15 μm. (B) Colocalisation analysis with a nuclear marker. Images are shown from left to right: DIC, NLSsfGFP (green), NfeIBP6(A20I)wrmScarlet (red), and the merged fluorescence image. The scale bar is 4 μm.

To examine the intracellular localisation of NfeIBP6(A20I) transgenic *C. elegans* expressing an NfeIBP6(A20I)–wrmScarlet fusion protein under a body wall muscle-specific promoter were generated. Confocal microscopy revealed that NfeIBP6(A20I)wrmScarlet was not uniformly distributed in the cytoplasm but instead accumulated in specific intracellular regions (Fig. 1A). Notably, strong fluorescence signals were observed in regions corresponding to the nucleus, although sequence motif analysis of NfeIBP6(A20I) did not reveal an obvious canonical nuclear localisation signal (Fig. S1). To further assess this localisation, worms coexpressing a nuclear marker (NLSsfGFP) were analysed. The red fluorescence of NfeIBP6(A20I)wrmScarlet partially overlapped with the green fluorescence of the nuclear marker, confirming its presence within the nuclear region (Fig. 1B). These observations indicate that NfeIBP6(A20I) exhibits a characteristic localisation pattern in *C. elegans* muscle cells, with distribution in both cytoplasmic and nuclear regions.

### 3.2 NfeIBP6(A20I) expression does not induce detectable toxicity

**Fig. 2:**
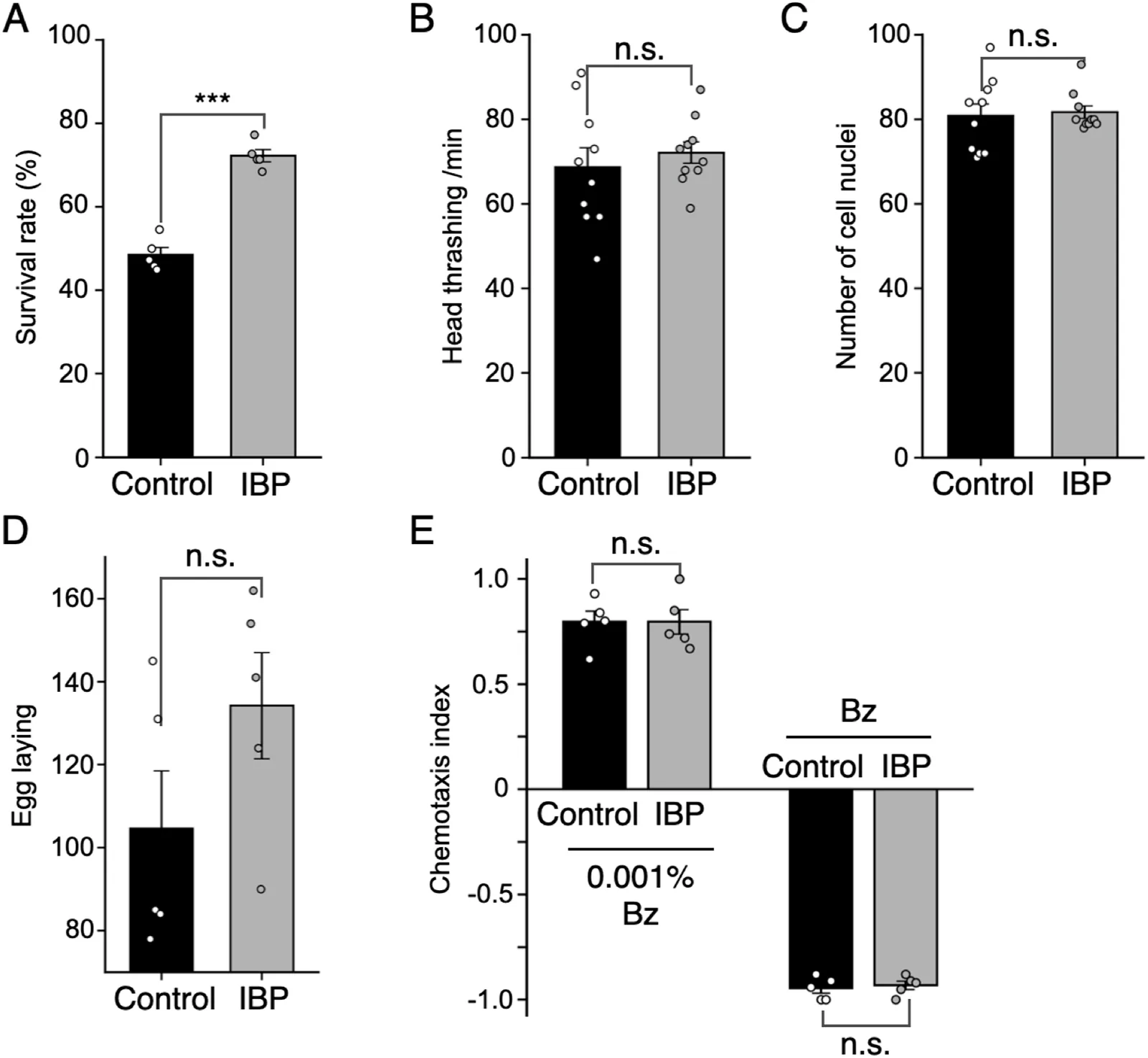
Physiological assessment of NfeIBP6(A20I)-expressing worms. (A) Survival rate at 5 °C. In each assay, n = 5 (28–39 animals per assay). (B) Thrashing assay, shown as head thrashings per minute. n = 10. (C) Number of muscle cell nuclei. n = 10. (D) Brood size, shown as the total number of eggs laid over two days. n = 5. (E) Chemotaxis indices in response to benzaldehyde (Bz) at low (0.001%) and high concentrations. In each assay, n = 5 (50–100 animals per assay).

To evaluate the physiological impact of NfeIBP6(A20I) expression and its nuclear accumulation, we first assessed the survival rate of the transgenic worms at 5 °C. Consistent with previous reports [18,20], compared with control worms, NfeIBP6(A20I)-expressing worms exhibited a significantly higher survival rate (Fig. 2A). We further examined potential toxicity under standard conditions by analysing thrashing behaviour, muscle cell number, brood size, and chemotaxis. Thrashing rates were 68.7 ± 4.5 min^−1^ in the Control group and 72.1 ± 2.5 min^−1^ in the IBP-expressing group, with no significant difference (Fig. 2B). The number of muscle cells was also comparable (80.8 ± 3.0 in Control worms vs. 81.7 ± 1.5 in IBP worms; Fig. 2C). Brood size over two days was slightly greater in IBP worms (104.6 ± 13.9 vs. 134.2 ± 12.8), but the difference was not statistically significant (Fig. 2D). In chemotaxis assays, the attraction of worms to low-concentration benzaldehyde (0.001%) (0.80 ± 0.05 vs. 0.80 ± 0.06) and the avoidance of high-concentration benzaldehyde (−0.95 ± 0.02 vs. −0.93 ± 0.02) were similar (Fig. 2E). These results indicate that NfeIBP6(A20I) expression does not cause detectable abnormalities under the conditions tested.

### 3.3 NfeIBP6(A20I) expression is associated with biased transcriptional changes

**Fig. 3:**
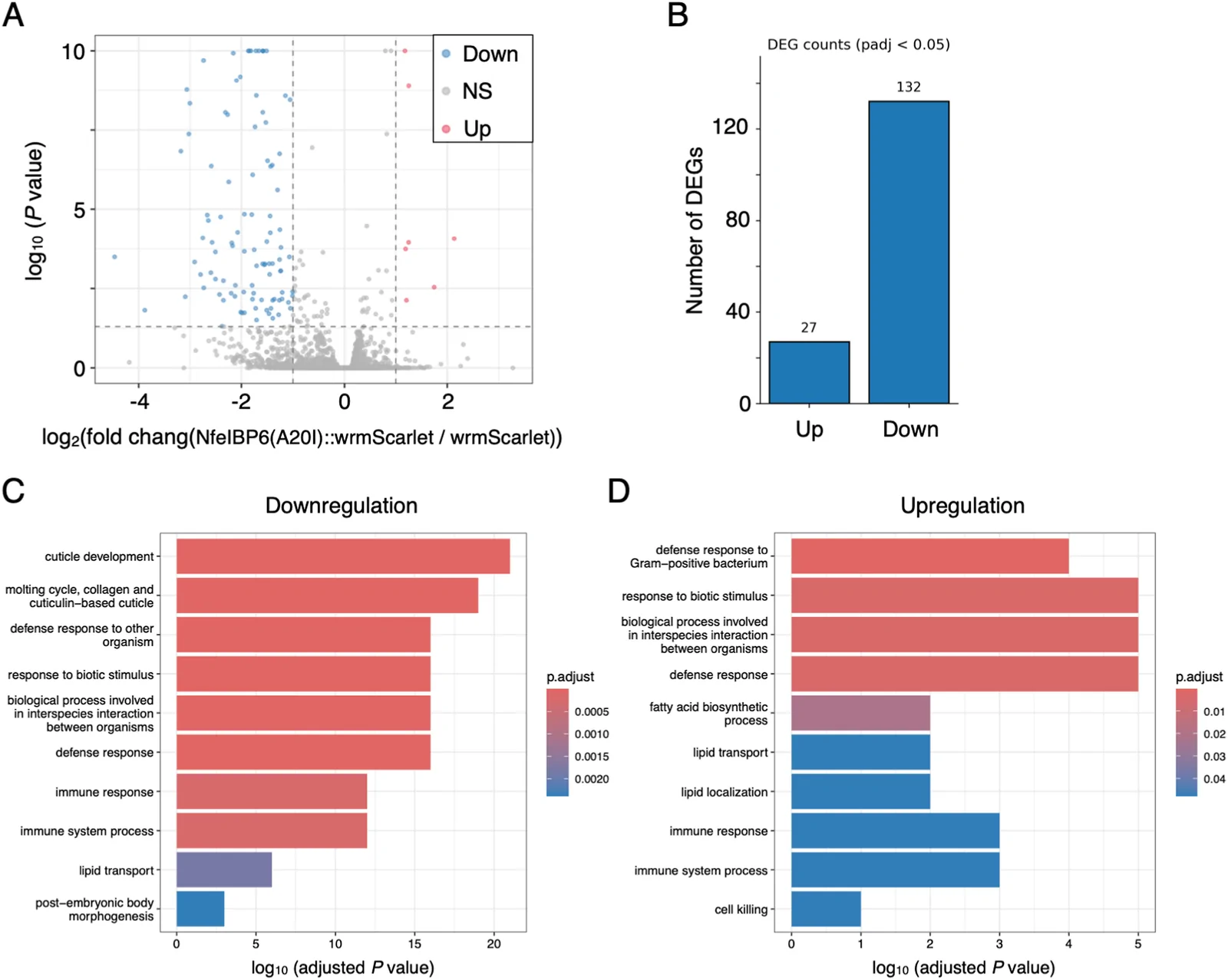
Transcriptional changes associated with NfeIBP6(A20I) expression. (A) Volcano plot showing genes that are differentially expressed between NfeIBP6(A20I)-expressing worms and control worms. Upregulated genes (red), downregulated genes (blue), and nonsignificant genes (grey) are indicated. (B) Number of differentially expressed genes (DEGs) (adjusted p value < 0.05). (C) Gene Ontology (GO) enrichment analysis of the downregulated genes, highlighting significant biological processes. (D) GO enrichment analysis of upregulated genes.

Given the nuclear localisation of NfeIBP6(A20I), its potential impact on gene expression was examined by RNA-seq analysis. Volcano plot analysis revealed multiple genes that were differentially expressed between NfeIBP6(A20I)-expressing worms and control worms (Fig. 3A). A total of 27 genes were upregulated, and 132 genes were downregulated, indicating a bias towards transcriptional downregulation (Fig. 3B). GO enrichment analysis revealed that the downregulated genes were significantly enriched in categories such as cuticle development, moulting cycle, defence response, and immune-related processes (Fig. 3C). In contrast, fewer genes were upregulated and showed enrichment in biological response and lipid-related processes (Fig. 3D). These results suggest that NfeIBP6(A20I) expression is associated with selective transcriptional changes, particularly those involving the downregulation of specific gene sets.

## 4 Discussion

The present study revealed that fish-derived NfeIBP6(A20I) is present within nuclear regions, in addition to the cytoplasm, when heterologously expressed in *C. elegans* muscle cells. This finding is noteworthy, given that many fish IBPs are generally considered to function extracellularly by preventing ice growth in body fluids. Our results combine high-resolution imaging with transcriptomic profiling and suggest that the functional repertoire of IBPs may extend beyond their well-characterised physical interaction with ice crystals to include previously unrecognised intracellular behaviour. The presence of NfeIBP6(A20I) within nuclear regions was unexpected, as sequence analysis did not reveal a canonical nuclear localisation signal. However, further investigation is required to determine whether its nuclear distribution is directly involved in regulating gene expression. For example, it would be useful to examine whether worms expressing NfeIBP6(A20I) fused to a nuclear localisation signal show similar transcriptional changes or physiological effects.

Previous studies have shown that the expression of NfeIBP6 variants enhances cold tolerance in *C. elegans* [20]. Among these variants, A20I has been reported to exhibit the highest ice-binding activity [23] and to confer a high level of cold tolerance on the worms. In addition, body-wall muscle cells are relatively large and are therefore suitable for detailed analysis of intracellular localisation by fluorescence microscopy. For these reasons, the present study primarily analysed worms expressing the A20I variant in the body-wall muscles. Interestingly, the WT (A20) also exhibited a localisation pattern similar to that of the A20I variant, suggesting distribution within the nucleus (Fig. S2). This finding suggests that the observed localisation pattern is not specific to the A20I variant and may represent a property shared by NfeIBP6 proteins. The expression and localisation of NfeIBP6 in other tissues or throughout the whole body were not examined in this study. Further studies are required to determine whether the observed nuclear distribution and associated transcriptional changes are specific to muscle cells or also occur in other tissues.

Because the NfeIBP6(A20I)wrmScarlet fusion protein has an estimated molecular mass of approximately 33 kDa, its entry into the nucleus through passive diffusion across the nuclear pore complex cannot be excluded [27,28]. Although the fusion protein was distributed in both cytoplasmic and nuclear regions and was not exclusively localised to the nucleus, the images suggested modest enrichment within the nuclear region. Passive diffusion alone may not fully explain this distribution, and additional mechanisms, including selective retention through interactions with nuclear components, non-canonical nuclear import pathways, or transport mediated by interacting proteins, may also be involved [29,30]. Similar nuclear localisation has been reported for certain plant IBPs [25], although its physiological significance remains unclear. The similar distribution of a fish-derived IBP within nuclear regions in an animal model raises the possibility that some IBPs interact with nuclear substructures or chromatin-associated complexes.

A further finding of this study is that NfeIBP6(A20I) expression was associated with a biased downregulation of gene sets, particularly those involved in cuticle development, the moulting cycle, and immune-related processes. In *C. elegans*, environmental stress is known to induce broad physiological and transcriptional responses, including changes in stress-responsive, immune-related, and structural pathways [[31], [32], [33], [34], [35]]. A previous study functionally analysed genes whose expression was altered in *C. elegans* with enhanced cold tolerance following drug treatment and reported that loss of the DAF-16 downstream genes *dod-22* and *dod-24* increased and decreased cold tolerance, respectively [36]. Because these genes are also involved in innate immune responses to bacteria [37], these findings suggest a functional link between the regulation of immune- and defence-related genes and cold tolerance. Therefore, the relative suppression of these pathways in IBP-expressing worms is of interest. One possible interpretation is that the cell-protective properties of NfeIBP6(A20I) help stabilise the intracellular environment and biological membranes, thereby reducing the extent to which endogenous stress-response pathways are engaged. Under such conditions, downstream responses such as cuticle remodelling may be less strongly induced. Although this interpretation remains speculative, it is consistent with the observed bias towards transcriptional downregulation.

From a physiological perspective, such an effect could be advantageous, as large-scale induction of stress‒response pathways is likely to impose a substantial metabolic cost. In this context, NfeIBP6(A20I) expression may contribute to the maintenance of cellular homeostasis without triggering overt developmental or behavioural abnormalities, as suggested by the results of the physiological assays performed here. However, at present, it remains unclear whether NfeIBP6(A20I) influences transcription more directly through interactions with nuclear components or whether the observed gene expression changes arise secondarily from altered intracellular conditions. Further studies, such as immunoprecipitation‒mass spectrometry (IP‒MS) or related interaction analyses, will be needed to identify the molecular partners of NfeIBP6(A20I) in vivo.

In conclusion, our study reveals a previously unrecognised intracellular aspect of IBP behaviour. The presence of NfeIBP6(A20I) within nuclear regions and its association with selective transcriptional changes suggests that the functional scope of IBPs may extend beyond conventional ice binding. These findings broaden the current understanding of IBP biology and provide a basis for future studies on the intracellular roles of IBPs.

## CRediT authorship contribution statement

**Saki Nakamura:** Formal analysis, Data curation. **Kazuki Ozawa:** Data curation. **Kotaro Ozaki:** Data curation. **Yoichi Shinkai:** Writing – review & editing, Writing – original draft, Data curation. **Masahiro Kuramochi:** Writing – review & editing, Writing – original draft, Visualization, Validation, Supervision, Software, Resources, Project administration, Methodology, Investigation, Funding acquisition, Formal analysis, Data curation, Conceptualization.

## Ethics declaration

This study was conducted in accordance with the ARRIVE (Animal Research: Reporting of In Vivo Experiments) guidelines. Ethics committee approval was not required because this study used *Caenorhabditis elegans*, an invertebrate species that is not subject to institutional animal ethics review under the applicable regulations and institutional guidelines.

## Declaration of competing interest

The authors declare that they have no known competing financial interests or personal relationships that could have appeared to influence the work reported in this paper.

## Data Availability

Data will be made available on request.
