# Supplementary material for: Nuclear localisation of a fish-derived ice-binding protein and related transcriptional changes in Caenorhabditis elegans
Source: Biochem Biophys Rep. 2026 Sep 2;47:102771. doi: 10.1016/j.bbrep.2026.102771 (PMC13571282; doi:10.1016/j.bbrep.2026.102771)
Supplement: Supplementary file 1 [file mmc1.docx]

**Supplemental Information:**

**Nuclear localisation of a fish-derived ice-binding protein and related transcriptional changes in *Caenorhabditis elegans***

Saki Nakamura^1^, Kazuki Ozawa^2^, Kotaro Ozaki^1^, Yoichi Shinkai^2^, Masahiro Kuramochi^1,3,*^

^1^Graduate School of Science and Engineering, Ibaraki University, Hitachi, 316-8511, Japan

^2^Cellular and Molecular Biotechnology Research Institute, National Institute of Advanced Industrial Science and Technology (AIST), Tsukuba 3058566, Japan

^3^Graduate School of Frontier Sciences, The University of Tokyo, Kashiwa, 277-8561, Japan

^*^Correspondence email: [masahiro.kuramochi.vw26@vc.ibaraki.ac.jp](mailto:masahiro.kuramochi.vw26@vc.ibaraki.ac.jp)

**Supplemental Information:**

MGESVVATQLIPINTALTPIMMEGKVTNPSGIPFAEMSQIVGKQVNTPVAKGQTLMPGMVKTYVPAK

**Fig. S1. Amino acid sequence of NfeIBP6(A20I).** The position of the A20I substitution is highlighted in yellow. The amino acid sequences of NfeIBP6 WT and the A20I variant were analysed for potential nuclear localisation signals using cNLS Mapper and NLStradamus. cNLS Mapper predicted neither a monopartite nor a bipartite NLS using a cut-off score of 2.0. NLStradamus produced no NLS prediction using Viterbi decoding, and no candidate region was detected using posterior decoding with a cut-off of 0.6. No candidate NLS was detected even when the posterior-decoding cut-off was lowered to 0.1. Thus, no canonical NLS was predicted in either NfeIBP6 WT or the A20I variant under the conditions used.

**Fig. S2. Intracellular localisation of NfeIBP6 WT (A20) in *C. elegans* body-wall muscle cells.** Representative confocal microscopy images showing the localisation of wrmScarlet-tagged NfeIBP6 WT (A20) expressed in body-wall muscle cells. White arrowheads indicate nucleolar regions. Scale bars, 4 µm.
